# Antioxidant Activities of 4-Methylumbelliferone Derivatives

**DOI:** 10.1371/journal.pone.0156625

**Published:** 2016-05-31

**Authors:** Yasameen K. Al-Majedy, Ahmed A. Al-Amiery, Abdul Amir H. Kadhum, Abu Bakar Mohamad

**Affiliations:** 1 Department of Chemical and Process Engineering, University Kebangsaan Malaysia (UKM), Bangi, Selangor 43000, Malaysia; 2 Fuel Cell Institute, University Kebangsaan Malaysia (UKM), Bangi, Selangor 43000, Malaysia; National Taiwan University, TAIWAN

## Abstract

The synthesis of derivatives of 4-Methylumbelliferone (4-MUs), which are structurally interesting antioxidants, was performed in this study. The modification of 4-Methylumbelliferone (4-MU) by different reaction steps was performed to yield the target compounds, the 4-MUs. The 4-MUs were characterized by different spectroscopic techniques (Fourier transform infrared; FT-IR and Nuclear magnetic resonance; NMR) and micro-elemental analysis (CHNS). The in vitro antioxidant activity of the 4-MUs was evaluated in terms of their free radical scavenging activities against 2,2-diphenyl-1-picrylhydrazyl (DPPH), Nitric oxide radical scavenging activity assay, chelating activity and their (FRAP) ferric-reducing antioxidant power, which were compared with a standard antioxidant. Our results reveal that the 4-MUs exhibit excellent radical scavenging activities. The antioxidant mechanisms of the 4-MUs were also studied. Density Function Theory (DFT)-based quantum chemical studies were performed with the basis set at 3-21G. Molecular models of the synthesized compounds were studied to understand the antioxidant activity. The electron levels, namely HOMO (highest occupied molecular orbital) and LUMO (lowest unoccupied molecular orbital), for these synthesized antioxidants were also studied.

## Introduction

**Fig 1:**
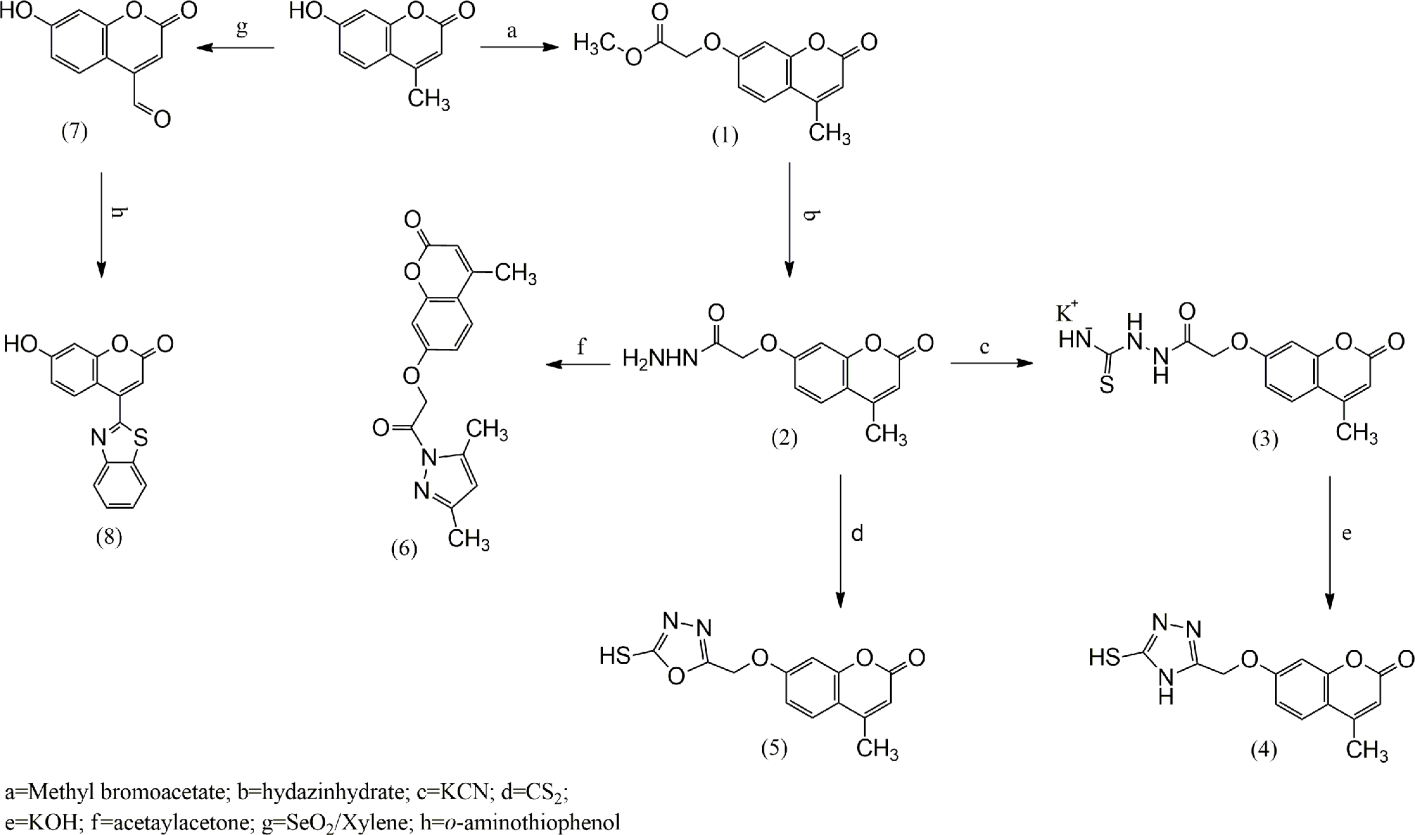
Synthesis of the 4-MU compounds

Umbelliferone derivatives (Us) are a family of coumarins that are recognized to have anti-inflammatory, antithrombotic, enzyme inhibitor and antioxidant properties [1–4]. The potency of various substituents on Us as antioxidants were deeply investigated by researchers, who found that the number and nature of the hydroxyl, methoxy or methyl substituents as electron-donating groups are the most important factors responsible for adjusting the antioxidant activities of Us. Generally, 4-MUs have excellent antioxidant properties based on the nature and number of substituted hydroxyl groups [5–9]. Coumarins inhibit the proliferation of various tumor cell lines, they retard the development of renal and prostate-carcinoma [10–12], in addition to prevent the repetition of melanoma [13]; Umbeliferons show cytotoxic impacts against the lung-carcinoma cell lines [14–16]. These compounds have undergo clinical attempts for therapy of the lymphedema subsequent breast-cancer therapy moreover therapy of kidney and lung carcinoma, that had been utilized jointly [17] in isolate and in combined with cimetidine, as an anti-neoplastic therapy [10,18, 19]. Based on the medicinal applications of Us and as a continuation of previous studies [20–23], we focus here on the design of our approach to increase the antioxidant activity based on a conjugated system and applied the theoretical studies to associate the antioxidant activities with the electronic structures. There was a good relationship between H atom abstraction and unpaired electron delocalization. To understand the relation between delocalization and radical reactivity, one can investigate the electron distribution in the HOMO and LUMO. The main aim of this study was to optimize the structures of all of the studied 4-MUs to explain the structure-antioxidant relationship. We had also been concerned with the calculation of antioxidant descriptors, such as the Dipole moment, Ionization potential (IP), Electron affinity (EA), Hardness (η), Softness (S), and Electronegativity (μ), for the 4-MUs and the standard compound (Trolox). In addition, we determined the preferred antioxidant mechanism and calculated the HOMO and LUMO energies and Band gap. The structure-antioxidant relationships of the synthesized antioxidants and Trolox were investigated using the DFT-based quantum chemical method together with the 3-21G basis set. Based on the obtained results, we conclude that the N-H, O-H, and S-H groups are responsible for antioxidant abilities. The quantum chemical calculations confirmed the high antioxidant activity of the 4-MUs. Initially, we used 4-MU as the starting material, and all of the synthesized 4-MUs are shown in Fig 1.

## Materials and Methods

### 2.1 General Information

All chemicals were supplied by Sigma-Aldrich (Selangor, Malaysia). The FT-IR spectra were obtained on a Nicolet 6700 FT-IR spectrophotometer (Thermo-Nicolet-Corp., Madison, WI, USA) in cm^−1^. The NMR spectra were recorded using an AVANCE III 600 spectrometer (Bruker, Billerica, MA, USA) using DMSO-d6 and are expressed in δ ppm. Elemental microanalysis was performed using an Elemental Vario El Iii, Carlo Erba 1108 Elemental analyzer.

#### 2.1.1 Synthesis of 4-MUs 1–8

Compounds (1–8), namely ethyl 2-((4-methyl-2-oxo-2H-chromen-7-yl)oxy)acetate (1), 2-((4-methyl-2-oxo-2H-chromen-7-yl)oxy)acetohydrazide (2), potassium (2-(2-((4-methyl-2-oxo-2Hchromen-7-yl)oxy)acetyl)hydrazinecarbonothioyl) amide (3), 7-((5-mercapto-4H-1,2,4-triazol-3-yl)methoxy)4-methyl-2H-chromen-2-one (4), 7-((5-mercapto-1,3,4-oxadiazol-2-yl)methoxy)4-methyl-2H-chromen-2-one (5), 7-(2-(3,5-dimethyl-1H-pyrazol-1-yl)-2-oxoethoxy)4-methyl-2H-chromen-2-one (6), 7-hydroxy-2-oxo-2H-chromene-4-carbaldehyde (7) and 4-(benzo[d]thiazol-2-yl)-7-hydroxy-2H-chromen-2-one (8), were synthesized using the procedures described by Al-Amiery in 2014 [24].

### 2.2 Antioxidant Activity

#### 2.2.1 DPPH radical scavenging activity assay

The DPPH radical scavenging activity assay [25] was performed with trivial adjustments. Various concentrations of the prepared compounds (250, 500, 750 and 1000 mg/mL) were used. The DPPH solution was prepared by dissolving 6.0 mg of this compound in 100 mL of the solvent (methanol). Then, 1 mL of each concentration was added to 2 mL of the prepared DPPH solution. Finally, the control was prepared by adding 1 mL of methanol to 2 mL of DPPH. Trolox was used as the standard. The mixture was shaken vigorously and incubated in the dark for 30 min. The absorbances of the resulting solutions were spectrophotometrically examined at 517 nm. The scavenging activities of each prepared concentration of the DPPH radicals were estimated using Eq (1).

$${DPPH}_{scavenginig\;effect}\;\%=A{}^{\circ}-\frac{A}{A{}^{\circ}}\times 100$$ (1)

where A° is the absorbance of the control reaction and *A* is the absorbance in the presence of the samples or standards. All samples were prepared and measured in triplicate.

#### 2.2.2 Ferric reducing/antioxidant power assay (FRAP)

The FRAP assay was performed as previously described [26]. The FRAP reagent was prepared from acetate buffer (pH 3.6), a 10 mM TPTZ solution in 40 mM HCl and a 20 mM iron (III) chloride solution in a ratio 10:1:1 (v/v), respectively. A total of 50 μl of the various concentrations of the prepared compounds were added to 1.5 ml of the FRAP reagent and mixed well. The concentrations were measured in three replicates. A standard curve was prepared using a similar procedure. The results were expressed as μmol Fe (II)/100 g of the synthesized compounds. Trolox was used as the standard.

#### 2.2.3 Nitric oxide radical scavenging activity assay

Aqueous solution Sodium nitroprusside at physiological pH generates nitricoxide spontaneously and interact with oxygen to yield nitrite ion that could be calculated utilizing reaction named Griess-Ilosvay [27]. In this study, Griess-Ilosvay reagent was adjusted utilizing naphthylethylenediaminedihydro chloride (0.10% w/v) rather than 1-naphthyl-amine (5.0%). Reaction mixture (3 mL) having sodium nitroprusside (10.0 mM, 2.0 mL) in addition to phosphate buffered saline (0.50 mL) and various concentrations of the prepared compounds (250, 500, 750, and 1000 _g/mL) or a standard solution (0.50 mL) that incubate at 25°C for 150.0 minute. After incubated, 0.50 mL of mixture having nitrite mixed with 1.0 mL of sulfanilic acid reagent that allows standing for 5.0 minutes to accomplishment diazotization process. Following, 1.0 mL of naphthylethylenediamine dihydrochloride (1.0%) was mixed, and left for 30 minutes. The absorbance had been measured at 540.0 nm versus the proper blank. The scavenging activities of each prepared concentration of the nitric oxide percentage were estimated utilizing Eq (1).

#### 2.2.4 Ferrous ions chelating activity

Chelating activity of the synthesized compounds toward Fe+2 was investigated as described in the reference [28]. Various concentrations of the prepared compounds (250, 500, 750, and 1000 _g/mL) or a standard solution (1.0 mL) were equally mixed with FeCl2 (0.05 mL), and ferrozine solution (0.2 mL), shaken and of incubation at room temperature. Absorbance was measured at 545 nm. EDTA had been utilized as positive control.

### 2.3 Quantum Studies

The molecular representation of the reference compound was plotted using ChemBioOffice 2010 software. All of the quantum chemical calculations were performed using the DFT methodology with the 3–21G basis set.

### 2.4 Statistical Analysis

The results were expressed as the means ± standard deviation, and the statistical significance of the differences was determined utilizing one-way analysis of variance (ANOVA) with the SPSS 17.0 statistical software program. Differences were considered significant at P < 0.05. The values are presented as the means ± SD (n = 3).

## Results and Discussion

### 3.1 Antioxidant activities

#### 3.1.1 DPPH radical scavenging activity assay

**Fig 2:**
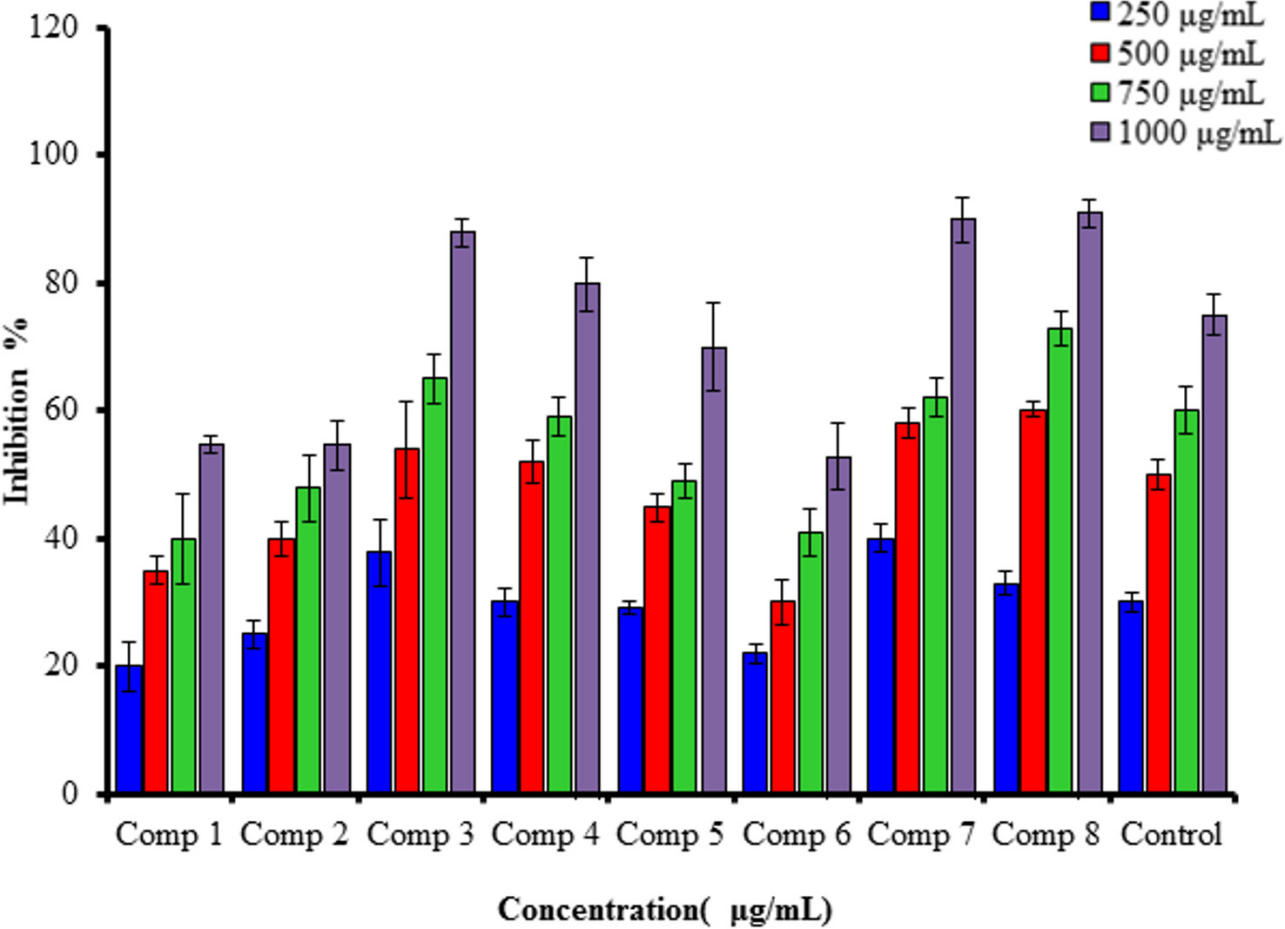
Antioxidant activity of different concentrations of the synthesized compounds (1–8) and Trolox in the DPPH radical scavenging assay.

The DPPH 1,1-diphenyl-2-picrylhydrazyl radical is a steady radical that could share an electron or hydrogen and transfer it to a stable compound. The methanolic DPPH solution showed a strong absorption band at 517 nm due to the single-electron (ode-electron), and if this solution was mixed with suitable reducing agents, the result would be pairing of the electron and a lack of color. The color is stoichiometrically reduced as the number of electrons taken up increases, and the decrease in the absorbance could be directly measured and compared with the standard (Trolox). Fig 2 shows the high DPPH radical scavenging activities compared with the Trolox standard.

#### 3.1.2. Proposed antioxidant mechanisms of 4-MUs 4 and 8

**Fig 3:**
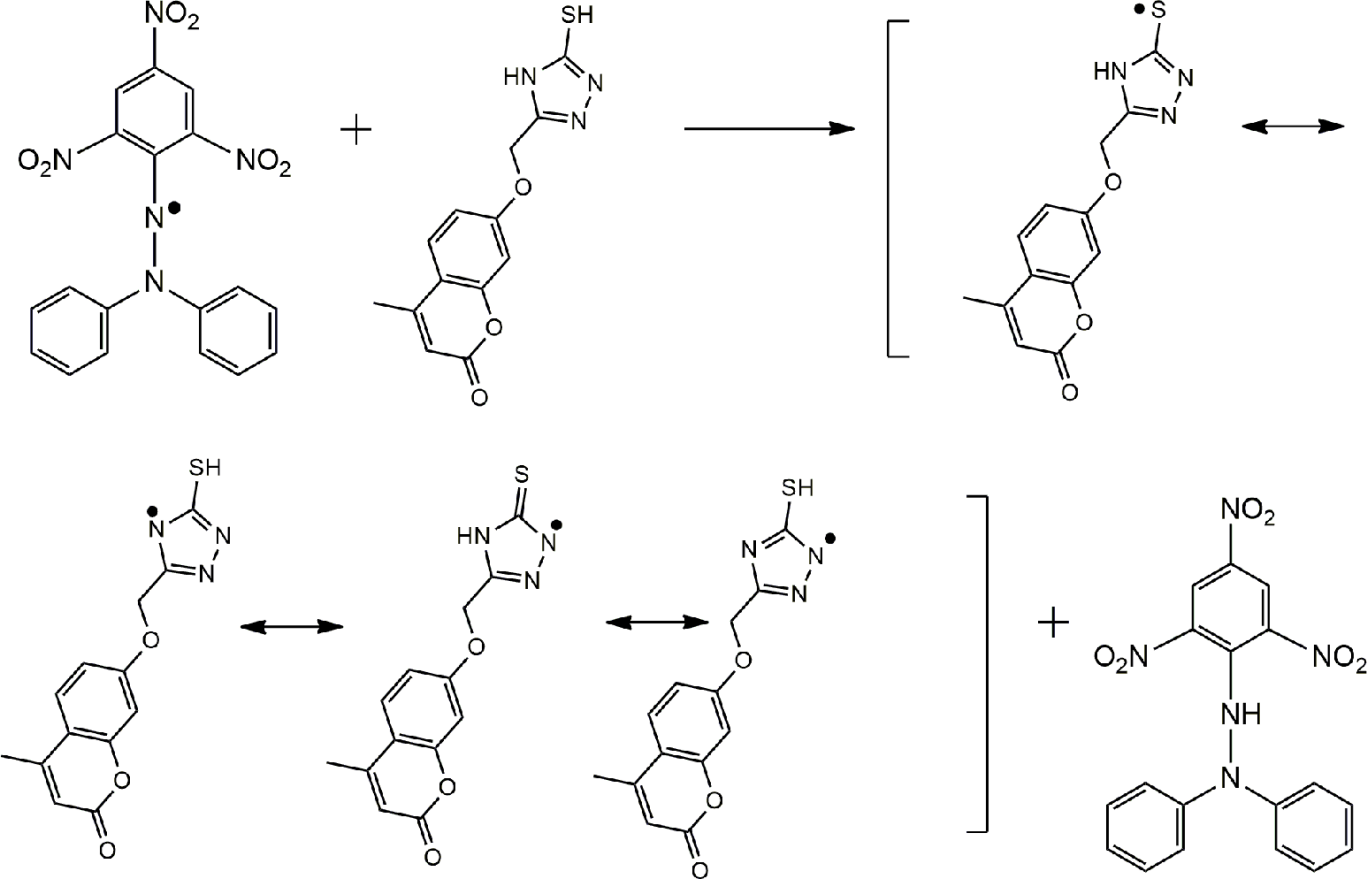
The reaction scheme between DPPH free radicals and compound 4.

**Fig 4:**
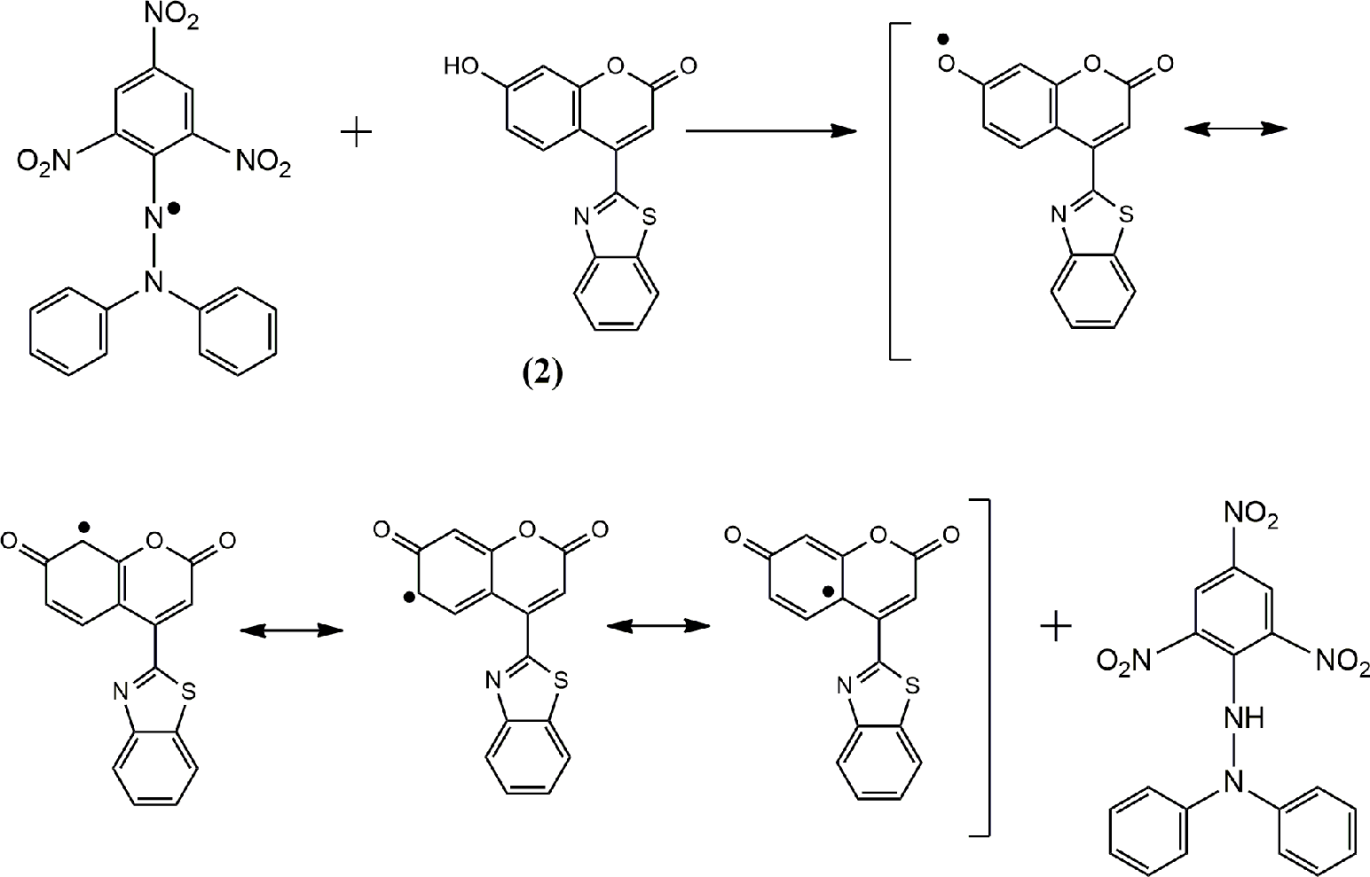
The reaction scheme between DPPH free radicals and compound 8.

Based on the chemical structures, we can assume that the antioxidant activities of the 4-MUs, as shown in Figs 3 and 4, depend on the hydrogen atoms of the hydroxyl and/or amino groups, which were under the impact of resonance and/or inductive effects. Resonance and inductive effects facilitate the reduction of the hydrogen group, making the molecule more stable. 4-MUs have scavenging activities due to the stability of the intermediate radicals of 4-MUs. The elimination of a hydrogen group from an amine or hydroxyl group may occur easily [29]. Heterocyclic residues and steric hindrance enhance the antioxidant activities [30, 31].

#### 3.1.3 Nitric oxide radical scavenging activity assay

**Fig 5:**
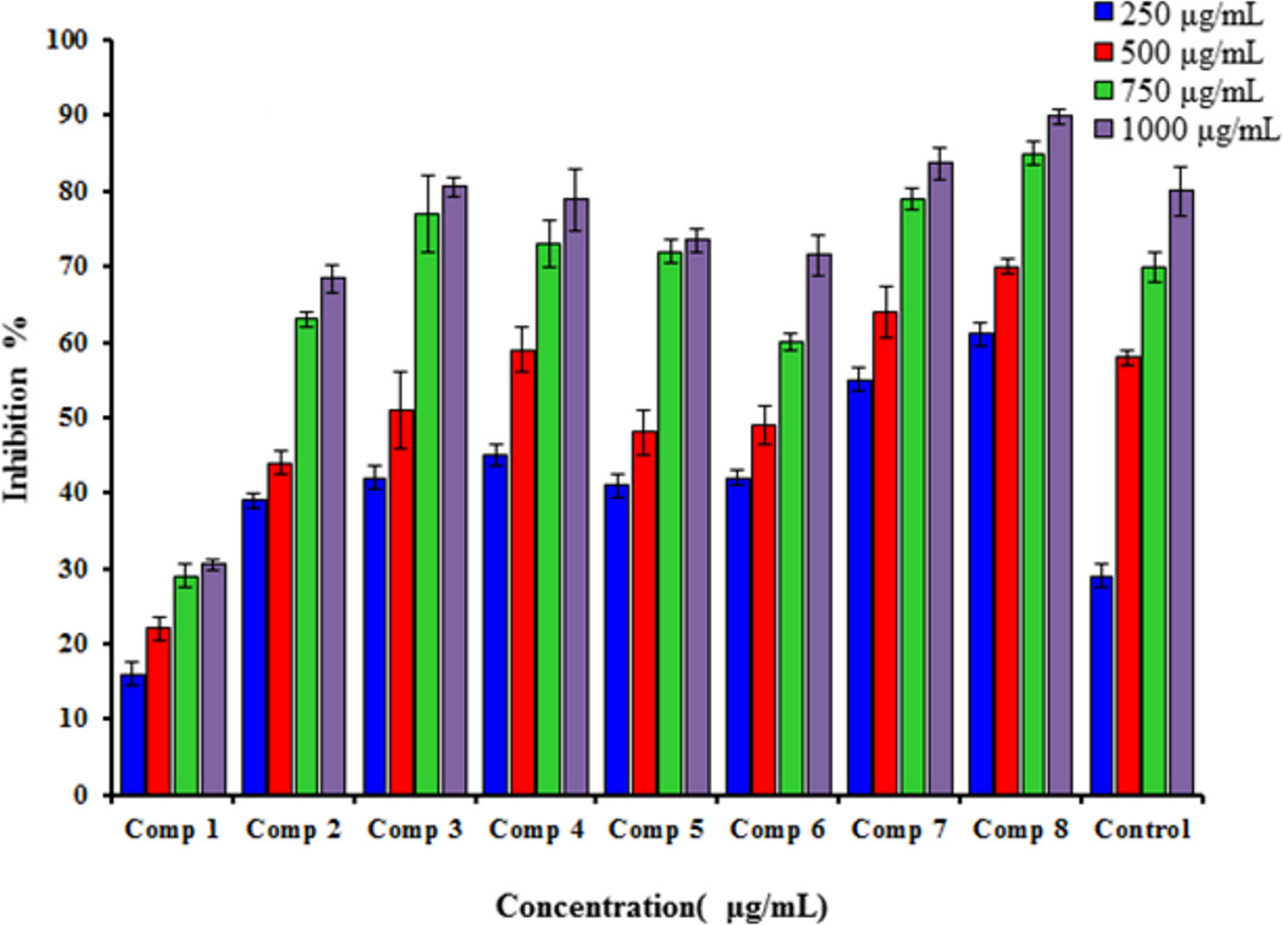
Antioxidant activity of different concentrations of the synthesized compounds (1–8) in the Nitric oxide radical scavenging assay

Nitric oxide radical (NO•), exclaimed as the " molecule of year" in 1992 by the Science journal [32], has a significant part in the control of various physiological and pathophysiological forms [33]. The Griess interaction is repeatedly utilized for evaluation of NO• generation by entire cells or enzyme [34]. Its applications to in-vitro limitation of NO• scavenging capacity is continual. For this situation, the nitric oxide staying after interaction with the sample is measured as nitrite. It is significant to confirm that nitrate can be formed, hence it ought to be diminished to nitrite before determination [35]. The derivative of azo chromophor was formed from nitrite after Griess reaction determined spectrophoto-metrically at 540 nm. Standard curve produced utilizing sodium nitrite and result was demonstrated as percentage change from control reaction. Compared with different strategies, is not direct, requiring the expansion of a several enzymatic reagents [36]. From Fig 5, it can be shown that compounds 3,4, 6 and 8 have the highest antioxidant activities.

#### 3.1.4 Chelating activity

**Fig 6:**
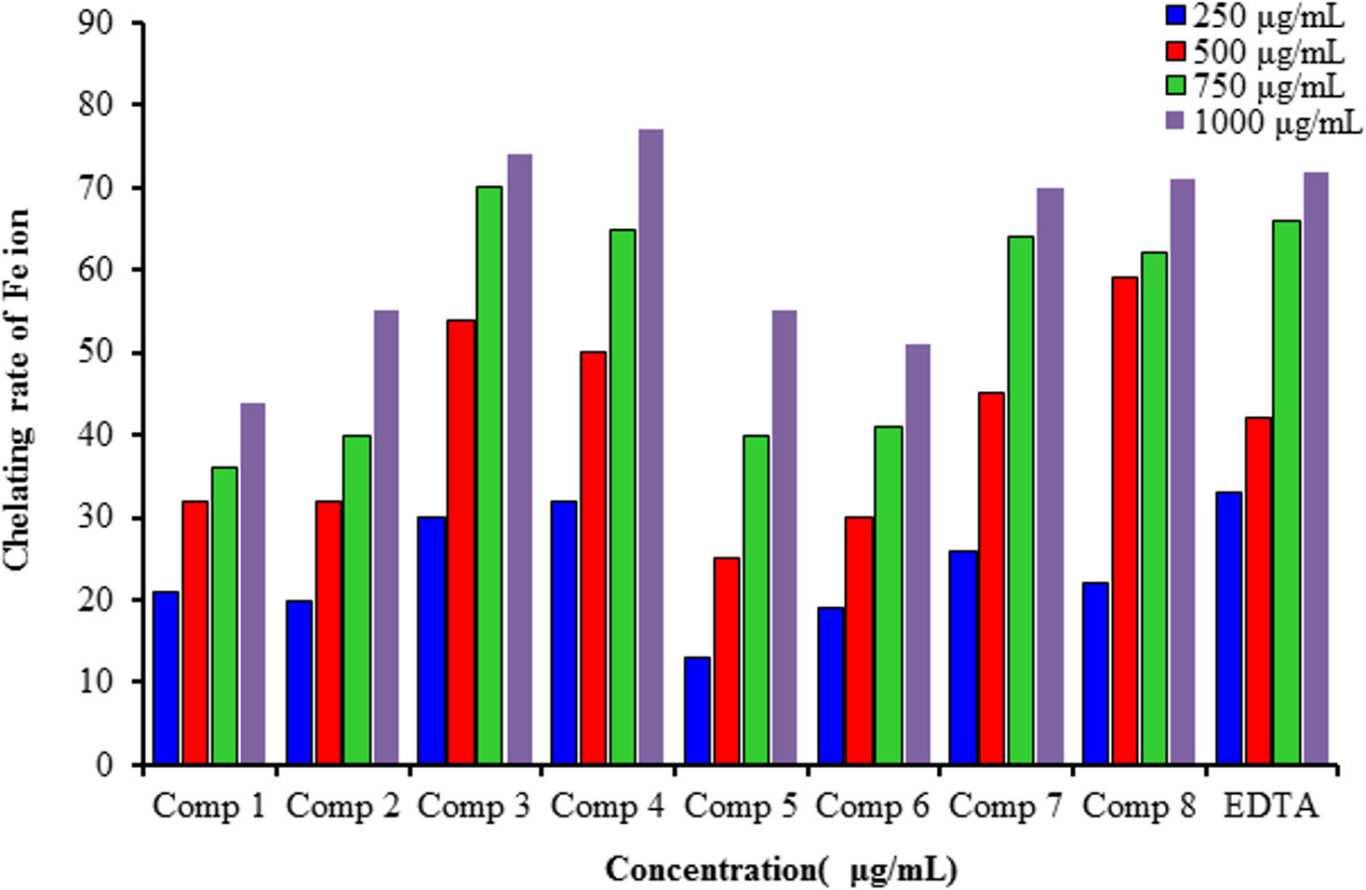
Fe^2+^ chelating activities of synthesized compounds 1–8

Amongst the significant mechanism of action of secondary antioxidant has been chelation of pro-oxidant metal. Iron enhances oxidation by expressible as catalyst of free-radical reaction. Iron has transfer single electron through replacements in oxidation state. Complexation of Iron with organic compounds decrease the pro-oxidant impact by reduce it redox potential and stabilize the oxidizing form of the Iron [37]. All the studied compounds had the ability to complexation with Iron. Iron complexation impacts of the synthesized compounds (1–8) were conditioned by concentrations and straight increased with the tested compound concentration raised. The relationship as in Fig 6, of compounds 1,2,5 and 6 to Fe+2 had been relatively-low as compare with EDTA. However the activity of compounds 3,4,7 and 8 were the like as the EDTA. superior effectiveness of the studied compounds 1–8 might also include the utilize of the synthesized compounds as iron chelators, similar to other compounds of that class [38,39].

### 3.2 Molecular Modeling Studies

**Fig 7:**
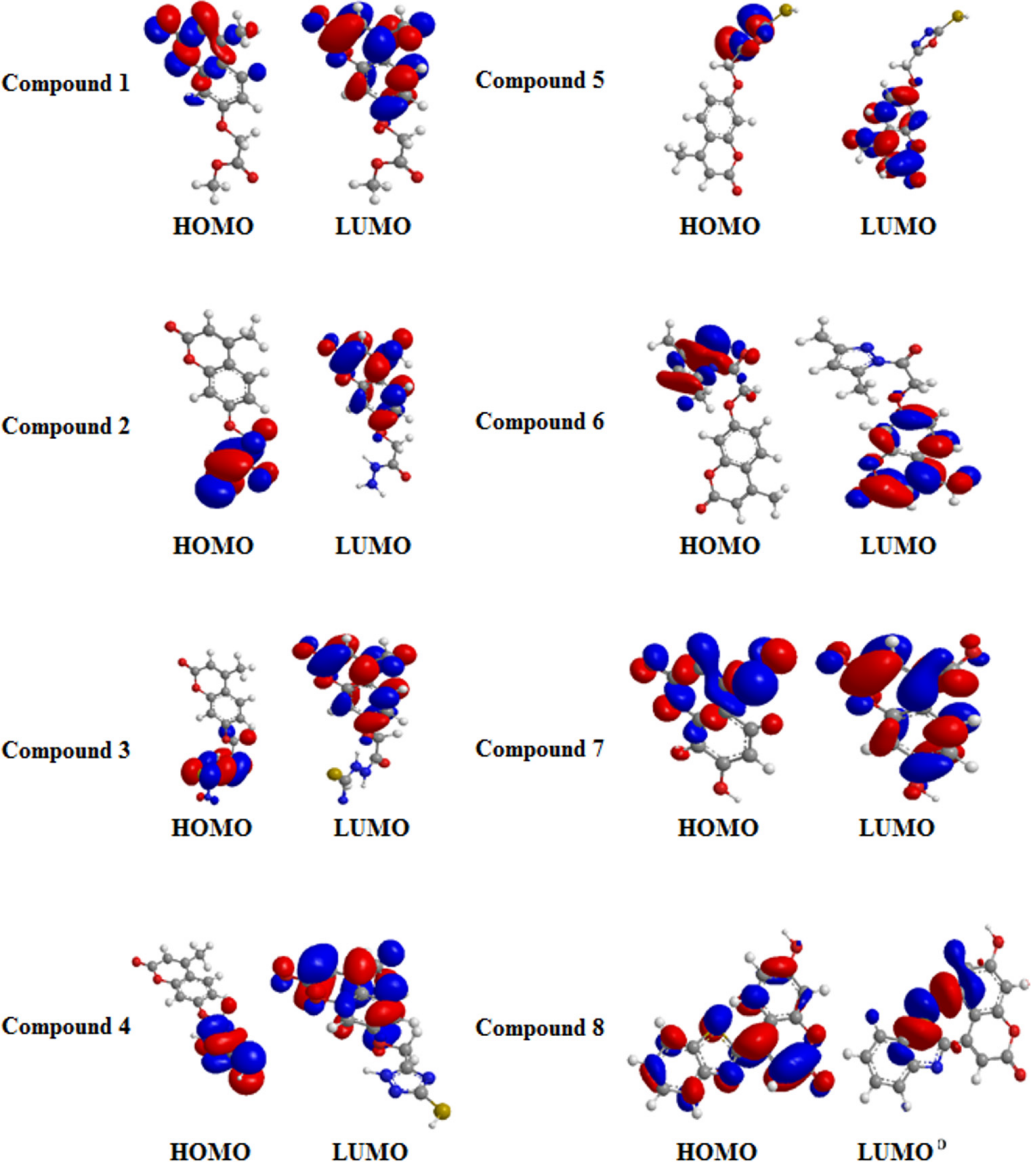
Highest occupied molecular orbital (HOMO) and the lowest unoccupied molecular orbital of compounds 1, 2, 3, 5, 6, 7 and 8.

To determine the scavenging activity and the electron levels (HOMO "highest occupied molecular orbital" and LUMO "lowest unoccupied molecular orbital") of the prepared scavengers (1–8), DFT-based quantum chemical investigations were performed with the basis set 3-21G. The EHOMO and ELUMO were reported as energies in eV (electron volt) values in Fig 7. The 4-MUs with higher scavenging activities could be established using the values of the HOMO and LUMO energies. In this study, we used the DPPH method, which obviously showed that the scavenging activities of 4-MUs 7 and 8 were higher than the others due to resonance effects of the aromatic ring and electron withdrawal. Theoretically, it was conceivable that the HOMO energy is a valid indicator for scavenging activity and that this scavenging activity was not based on LUMO energy. The differences in the scavenging activities of the 4-MUs (1, 2, 3, 5, 6, 7 and 8), as represented by the HOMO values, essentially refer to the delocalization of pi-electrons, leading to the stability of the radicals acquired after hydrogen abstraction such that the delocalized pi-electrons in the MUs (1, 2, 3, 5, 6, 7 and 8) (Fig 7) also occur in the corresponding radical. The HOMO electron density could be considered to understand the relation between the pi-electrons and scavenging activity [40].

The HOMOs (highest occupied molecular orbitals) of 4-MUs (1–8) are delocalized over the entire molecules, which agree with the unshared electrons. The spin densities of the free radicals that had been generated from 4-MUs (1–8) were compared. Increased delocalization indicates that the generation of free radicals was easier. The spin density seems to be slightly more delocalized for the radicals created from 4-MUs (7 and 8) than for the other 4-MUs. The HOMO energies of the 4-MUs (1–8) and the reference compound are computed as -12.084 eV, -7.051 eV, -7.401 eV, -9.748 eV, -9.735 eV, 9.788 eV, 11.380 eV and -5.279 eV. Moreover, the LUMO energies for the 4-MUs (1–8) and reference compound are estimated as -4.062 eV, 3.876 eV, -3.883 eV, -3.875 eV, -3.875 eV, -3.872 eV, -4.398 eV and -3.983 eV. The energy gaps for the 4-MUs (1–8) and the control were recorded as 8.022 eV, 3.715 eV, 3.118 eV, 5.837 eV, 5.806 eV, 5.916 eV, 5.98 eV, and 1.296 eV due to the shift of absorption toward the blue region. Delocalization of the heterocyclic ring and carbonyl group for the 4-MUs 7 and 8, respectively, were responsible for the variations between the HOMOs and LUMOs of the 4-MU scavengers (1–8). A comparison of the potential according to the band gaps for 4-MUs (1–8) and control clearly showed that the highest band gap was observed for the control (reference), followed by 4-MUs 7 and 8, and this is highly compatible with the results of the experiment shown in Fig 2. The Dipole moment values of the 4-MUs (1–8) and control indicated that they are polar compounds and may be soluble in polar solvents. The estimated IP (Ionization potential) could provide an understanding of the initial energy required to release an electron from the compounds [41], which implied an inverse relation between the scavengers and IP (Eq 2).

$$\text{IP}=-{\text{E}}_{\text{HOMO}}$$ 2

EA (Electron affinity) is the amount of energy launched when an electron is absorbed by a molecule (Eq 3). Higher EA values allow the compound to easily absorb electrons. In other words, the compound has a positive relation with the antioxidant activity.

$$\text{EA}=-{\text{E}}_{\text{LUMO}}$$ 3

η (Hardness) is charge transfer resistance, and S (softness) is the measure of the capacity of an atom to receiving an electron (Eqs 4 and 5, respectively).

$$\eta =-\frac{1}{2}({\text{E}}_{\text{HOMO}}-{\text{E}}_{\text{LUMO}})$$ 4

$$\text{S}=-\frac{2}{\left({\text{E}}_{\text{HOMO}}-{\text{E}}_{\text{LUMO}}\right)}$$ 5

μ (electronegativity) is defined as the capacity to attract electrons (Eq 6) in the chemical bond.

$$\mu =-\frac{1}{2}({\text{E}}_{\text{HOMO}}+{\text{E}}_{\text{LUMO}})$$ 6

Table 1 obviously shows the potential values of the studied parameters, and these parameters could support the high scavenging activities. The theoretical parameters (using DFT, with the basis set 3-21G) and experimental findings were compared. A good correlation between the experimental and calculated data was observed. In addition, the HOMOs and LUMOs of the 4-MUs (1–8) were investigated using corresponding methods with the 3-21G basis set. The calculated HOMO-LUMO energies were used to calculate some of the properties of title molecule.

**Table 1:** The Electronic Properties of antioxidants 1–8 were obtained using the DFT method with the 3-21G basis set.

Parameters	Comp. 1	Comp. 2	Comp. 3	Comp. 4	Comp. 5	Comp. 6	Comp. 7	Comp. 8	Trolox
**IP; eV**	12.084	7.051	7.401	9.748	9.730	9.877	11.380	5.279	10.215
**EA; eV**	4.062	3.876	3.883	3.875	3.875	3.872	4.398	3.983	0.704
**η**	4.011	1.812	1.559	2.918	2.403	2.958	2.990	0.658	5.107
**S**	0.249	0.538	0.641	0.342	0.344	0.388	0.334	1.543	0.210
**μ**	8.073	5.463	5.642	6.811	6.802	6.874	7.889	4.631	5.459
**EHOMO**	12.084	7.051	7.401	9.748	9.730	9.877	11.380	5.279	10.215
**ELUMO**	4.062	3.876	3.883	3.875	3.875	3.872	4.398	3.983	0.704
**Band gap**	8.022	3.715	3.118	5.837	5.806	5.916	5.98	1.296	9.509

### 3.3 FRAP assay

**Fig 8:**
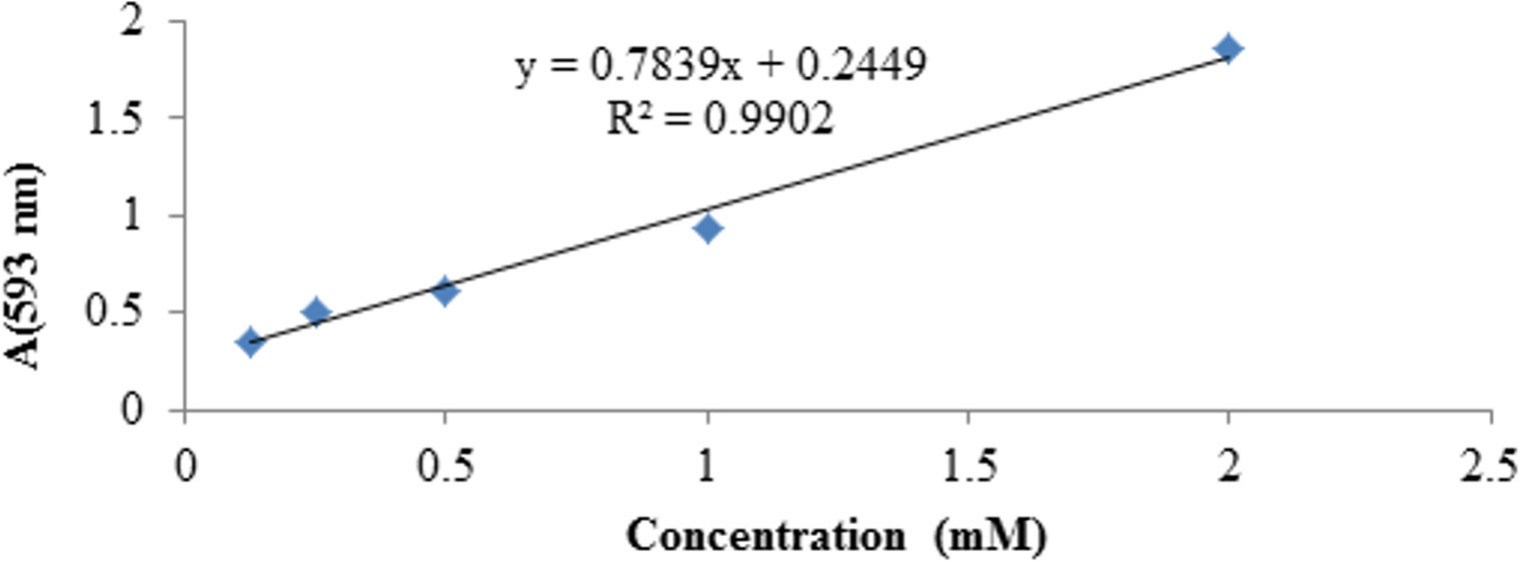
Linearity of the FRAP assay (dose–response line) for standard solutions.

The FRAP assay measures the capability of a molecule to diminish the ferric 2,4,6-tripyridyl-s-triazine complex to the colored ferrous-complex [42]. FRAP significances are obtained by comparing the absorbance shift to 593 nm in the examined reaction mixtures with that containing a recognized concentration of ferrous-ions. The linearity of the FRAP assay for standard solutions is shown in Fig 8. The results of the FRAP assay are reported in Table 2. The scavenger activities were expressed as the concentrations of antioxidants having a ferric-reducing ability equivalent to that of 1 mM of FeSO_4_, and the results showed that three of the 4-MUs (3, 7 and 8) exhibited higher antioxidant capacity.

**Table 2:** Antioxidant activity of (1–8) compounds using the FRAP assay.

Compounds	Inhibition (%)
1	50%
2	20%
3	75%
4	55%
5	40%
6	25%
7	73%
8	77%

## Conclusions

4-Methylumbelliferons (4-MUs) were successfully synthesized and characterized using spectroscopic techniques (FT-IR and NMR) and micro-elemental analysis (CHNS). The antioxidant activities were evaluated by DPPH and FRAP assays, and the results indicated that these compounds have good scavenging activities. The antioxidant mechanisms of the synthesized 4-MUs were also studied. DFT-based quantum chemical studies were performed with the basis set 3-21G, and molecular modeling was performed for the synthesized 4-MUs to understand their antioxidant activities. The electron levels, namely the HOMO (highest occupied molecular orbital) and the LUMO (lowest unoccupied molecular orbital), were also studied. We were also concerned with the calculation of the following antioxidant descriptors for the synthesized 4-MUs and the reference compound: Dipole moment; Ionization potential (IP), Electron affinity (EA), Hardness (η), Softness (S), and Electronegativity (μ).
